# Potential Applications of Magnesium-Based Polymeric Nanocomposites Obtained by Electrospinning Technique

**DOI:** 10.3390/nano10081524

**Published:** 2020-08-04

**Authors:** Adrián Leonés, Marcela Lieblich, Rosario Benavente, José Luis Gonzalez, Laura Peponi

**Affiliations:** 1 Instituto de Ciencia y Tecnología de Polímeros (ICTP-CSIC), C/Juan de la Cierva 3, 28006 Madrid, Spain; aleones@ictp.csic.es (A.L.); rbenavente@ictp.csic.es (R.B.); 2 Interdisciplinary Platform for “Sustainable Plastics towards a Circular Economy” (SUSPLAST-CSIC), 28006 Madrid, Spain; 3 Centro Nacional de Investigaciones Metalúrgicas (CENIM-CSIC), 28040 Madrid, Spain; marcela@cenim.csic.es (M.L.); jlg@cenim.csic.es (J.L.G.); 4 CIBER-BBN, 28040 Madrid, Spain

**Keywords:** electrospinning, magnesium nanoparticles, polymer nanocomposites, biomedical applications, energetic devices, environmental applications, post–processing, thermal treatments, industrial catalysis, antibacterial agent

## Abstract

In the last few decades, the development of new electrospun materials with different morphologies and advanced multifunctional properties are strongly consolidated. There are several reviews that describe the processing, use and characterization of electrospun nanocomposites, however, based on our knowledge, no review on electrospun nanocomposites reinforced with nanoparticles (NPs) based on magnesium, Mg-based NPs, are reported. Therefore, in the present review, we focus attention on the fabrication of these promising electrospun materials and their potential applications. Firstly, the electrospinning technique and its main processing window-parameters are described, as well as some post-processing methods used to obtain Mg-based materials. Then, the applications of Mg-based electrospun nanocomposites in different fields are pointed out, thus taking into account the current trend in developing inorganic-organic nanocomposites to gradually satisfy the challenges that the industry generates. Mg-based electrospun nanocomposites are becoming an attractive field of research for environmental remediation (waste-water cleaning and air filtration) as well as for novel technical textiles. However, the mayor application of Mg-based electrospun materials is in the biomedical field, as pointed out. Therefore, this review aims to clarify the tendency in using electrospinning technique and Mg-based nanoparticles to huge development at industrial level in the near future.

## 1. Introduction

In the last decades, the development of new nanocomposites with different morphologies and advanced multifunctional properties are strongly consolidated [1,2,3,4]. A wide variety of nanocomposites are synthetized and characterized in order to try to solve human needs in different fields, such as food packaging [5], biomedical devices [6] or environmental remediation [7] among others. The increasingly high requirements that these materials should fulfill are the driving force for scientists to focus their efforts on them and on advanced technologies needed for their production [8].

**Figure 1:**
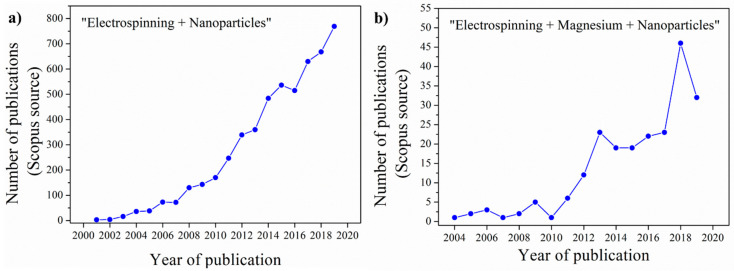
Number of publications per year looking for keywords: (**a**) “electrospinning” + “nanoparticles” and (**b**) “electrospinning” + “magnesium” + “nanoparticles” (Scopus Source).

Among all the techniques available to obtain polymeric materials at nanolevel, electrospinning has rapidly emerged in the last years, due to its suitability to produce polymeric nanofibers in relatively simple manner and at low cost [9,10]. This becomes manifest by the continuously increasing number of scientific publications based on the use of this technique. There were more than 700 documents in a Scopus search of the last 20 years, as indicated in Figure 1, where it is worth noting the strong increment of the publication number of scientific papers published until 2019 looking for keywords “electrospinning” and “nanoparticles” (Figure 1a). Polymeric electrospun nanofibers reinforced with nanoparticles (NPs) present a huge number of potential applications [10] thanks to the fact that they combine the flexibility and porosity of electrospun polymeric mats with the functional properties of NPs [11]. There are several reviews that describe the processing, use and characterization of nanomaterials and electrospun-based nanocomposites [12,13]. Similarly, several post-treatment processes have been reported to enhance the properties of electrospun materials [14]. However, based on our knowledge, no review on electrospun nanocomposites reinforced with nanoparticles based on magnesium, Mg-based NPs, are reported. 

Additionally, a large number of different NPs has been studied with very promising results in different applications: from biomedicine to electronic, optical and sensor applications [15,16,17]. Among them, magnesium Mg-based NPs, on which we focus this review, are considered very interesting due to the specific characteristics of this alkaline metal. Even if Mg is the fourth most common element in the Earth and an essential element in the human body as osteoconductivity material, Mg combines low density, high specific strength, stiffness, electrical conductivity, heat dissipation and absorption of vibrations [18]. Additionally, it has been reported to be effective against bacterial infections [19] and to play an important role for bone tissue engineering [20]. These properties make it very advantageous to study nanocomposites reinforced with Mg-based NPs and in particular, woven no-woven Mg-based materials obtained by electrospinning marks a recent line of research in the scientific panorama. Actually, in the last 10 years, the number of publications on electrospinning with magnesium-based nanoparticles has strongly increased (Figure 1b). However, based on our knowledge, no review on electrospun nanocomposites reinforced with Mg-based NPs are reported. Therefore, in the present review, we focus the attention on the fabrication of these promising electrospun materials and their applications. Firstly, the electrospinning technique and the main processing window-parameters are described, as well as some post-processing methods commonly used. Finally, the applications of the newest Mg-based electrospun nanocomposites in different fields are pointed out.

## 2. Electrospinning Technique and Post-Processing Methods

In 1934 Formhals patented an experimental method to create artificial wires using electrostatic force [21], although, this effect was first observed in 1897 by Rayleigh and investigated more widely by Zeleny (1914) [22]. When used to spin nanofibers, this procedure is called electrospinning. Larrondo and Manley proved in 1981 [23] that continuous filaments of rapidly crystallizing polymers could be spun from the melt using an electric field, and by 1996, Reneker and Chun studied the ability of some polymer solutions to be electrospun [24]. In the last years, a great quantity of scientific works on electrospinning have been recognized in the field of agriculture [25], filtration [26], tissue engineering [27] and packaging [28] among others.

**Figure 2:**
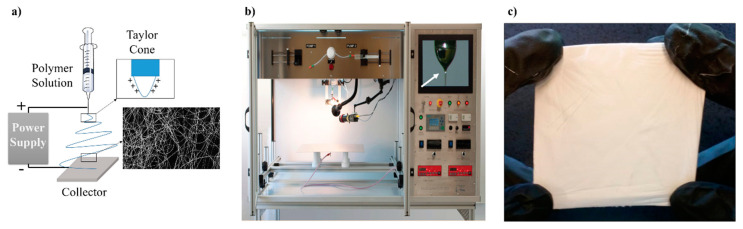
(**a**) Schematic representation of electrospinning setup, (**b**) electrospinning co-axial equipment where Taylor cone could be appreciated (indicated with a white arrow) and (**c**) randomly oriented woven no-woven poly (L-lactic acid) (PLA) electrospun nanofiber mat.

Usually, electrospinning equipment consists in a syringe holding the polymer solution with its needle connected to a direct current (DC) voltage supply (kV range) while another electrode is connected to the collector so that charges are induced in the solution. When the amount of charges is critical, the electric field promotes the polymer drop resulting in the formation of a Taylor cone (Figure 2b). Then, the polymer jet crosses the electric field to a grounded collector where nanofibers are deposited and collected as woven no-woven mats (Figure 2c). Unlike conventional fiber-spinning techniques, fibers in the nanometer range can be produced by this method easily and at low cost. For this reason, electrospinning has been established as a promising process for development of new materials at nano scale.

Electrospinning process can be influenced by three different parameters which may be classified into chemical parameters (molecular weight, viscosity, surface tension, …), processing conditions (applied voltage, distance, flow rates, …) and ambient conditions (temperature, humidity, …).

In particular, polymer solution and the molecular weight of the polymers are found to be ones of the most significant in the formation of fibers. For a suitable electrospinning process, a sufficient molecular weight of polymer is required in order to increase the entanglement between polymer chains which are crucial for the continuity of the jet formed during the electrospinning process. Thus, many authors have outlined the entanglement of chains as the main parameter in fiber formation. For example, Shenoy et al. [29] studied the transition from electrospraying to electrospinning due to the variation in the entanglements and the molecular weights of polymers used. 

The length of the polymer principally affects the viscosity of the solution that has to present a concentration high enough to cause polymer entanglement, but not so high when the solution viscosity avoids polymer motion induced by the electric field. Moreover, viscosity that is too high will make difficult to pump the solution through the needle and may dry the solution before the Taylor cone formation. Furthermore, high molecular weight provokes an increase in viscosity solution which has been associated with the production of larger diameter fibers [30] while too low concentrated solutions yield fibers with beads. In order to obtain continuous fibers without defects it is necessary to find an ideal concentration which will depend on the polymer used [31]. The relationship between molecular weight and viscosity is also manifest in the distribution of fiber diameters. A high concentration of polymer implies an increase in the viscosity so a secondary jet could emerge from the main electrospinning jet resulting in fibers with irregular diameters producing a bimodal distribution of fiber sizes [31,32].

Although viscosity has an important role in the formation of fibers, the initiation of electrospinning depends on another important factor which is the surface tension. The charged solution has to overcome the surface tension in order to form the polymer jet [33]. If the solution has low viscosity, a high tendency for the solvent molecules to form a spherical shape (beads) will be observed due to the action of surface tension. The solution electrical conductivity is another key parameter influencing the fibers morphology, affecting the solution ability to flow when an electrical field is applied to it. Thus, the formation of a single or multi jet can be facilitated due to electrical conductivity which can increase with the addition of salts and ions [31].

The chemical properties of solvents have to be also taken into account with their solubility parameters [34]. The most common solvents used in electrospinning process are: N,N-dimethylformamide (DMF), dichloromethane (DCM), tetrahydrofuran (THF), chloroform and methanol [9,35]. In order to reduce bead formation, different solvents are usually combined with the aim of increasing the dielectric constant of the solution. Especially common is the use of DMF, for example Hsu et al. [36] reported how the addition of DMF to the solution increased the deposition rate and dramatically reduced the average fiber diameter. It is necessary to emphasize that the addition of a solvent with the objective of increasing the dielectric constant, will affect the solubility of the polymer and therefore its electrospinnability.

The parameters of the electrospinning technique need also to be optimized to determine the processing conditions. Applied voltage, flow rate and type of collector, among other variables, strongly affect the morphology of the fibers. Firstly, voltage applied will create the electric field responsible for the starting of the electrospinning process. Then, the charges induced into the solution have to overcome the surface tension to form the Taylor cone. For it, a voltage in the range of kV is usually applied between positive and negative electrode. The influence of the applied voltage on the diameter of the fibers has been widely studied: Sohrabi et al. [37] investigated the effect of electric field and distance-voltage combination on the average diameter and size distribution of nanofibers. Buchko et al. [38] reported that applied fields could influence the morphology of the fibers, creating a variety of new shapes on the surface. Deitzel et al. [31] found a tendency of beads formation with high voltage values during the electrospinning process.

Flow rate of solutions is also decisive because it will determine the amount of polymer available during the process. The stability of the Taylor cone is associated to a particular flow rate for a given voltage so that varying these parameters beads-fibers are obtained. An increase in fiber diameters is also observed when the flow rate increases. However, there is a maximum flow rate that can be used during electrospinning process [39].

For a successful electrospinning process, the application of an electric field between the needle and the collector is required. Varying the type of collector, a wide variety of fibers with different morphology can be obtained. Usually, an aluminum foil (electrically grounded) is placed on the surface of the collector to accumulate the electrospun fibers due to its manageability. Also, the texture of electrospun mats can be varied using a patterned collector suitable for a specific application. For instance, Xiao et al. [40] have developed multi-nanostructured poly (L-lactic acid) (PLA) fibrous matrices patterned to manipulate biomolecule distribution and functions.

Depending on whether the collector works statically or moving, the morphology of the fibers will change. A rotating collector has been used to obtain aligned fibers while a static collector is required to fabricate random distributions of fibers. The speed of collector also affects the evaporation process of solvent. Wannatong et al. [41] related the speed of the rotating collector with evaporation of solvent and reported that when the rotating speed increased, the evaporation of DMF increased, resulting in fibers with no solvent trapped into the electrospun network. 

In addition, ambient conditions strongly affect the formation of fibers. With high humidity, condensation phenomenon will influence the fiber morphology. Some authors have applied this feature to obtain hollow and porous fibers via electrospinning. For example, Huang and Thomas [42] obtained micron-sized fibers with controlled surfaces and internal morphologies using specific solvents and varying the mechanisms in order to achieve hollow and porous fibers of PLA with high surface-area ratio for oil–water separation. In any case, the environmental conditions during electrospinning process have been poorly investigated. Some interaction between the composition of the air and the formation of fibers has been reported by Baumgarten [43] in 1971 but further studies should be carried out. In order to summarize the multiple factors affecting the electrospinning process, in Table 1 the most relevant ones and their effects on the fiber morphology are summarized.

**Figure 3:**
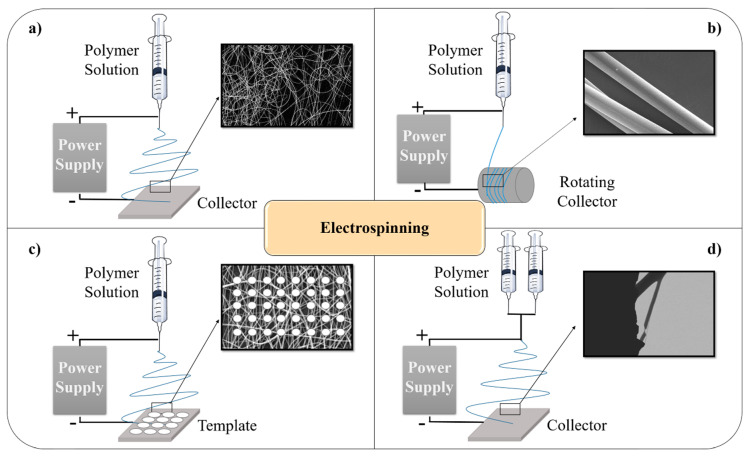
Widely used electrospinning setups (**a**) randomly oriented, (**b**) rotating collector, (**c**) template electrospinning and (**d**) co-axial electrospinning.

Moreover, in order to further control the electrospinning process and thus to tailor the structures of resultant fibers, the classical setup (Figure 3a) has also been modified. Moreover, for many applications, in which is necessary to control the orientation of nanofibers and their alignment, a rotating collector can be used. Several research works have been carried out involving the use of a rotating drum (Figure 3b) in different fields such as developing new material for high-technologies [44], piezoelectric materials [45] or medical applications [46]. Moreover, template electrospinning consists in a collector with geometrical pattern configuration which deeply influences the deposition of electrospun nanofibers (Figure 3c). The design of the template of the collector widely influences the properties of the final woven no-woven electrospun mat produced obtaining electrospun fiber mats with diverse mechanical properties, pore size or thickness [47]. Furthermore, co-axial electrospinning can produce core-shell fibers when two different polymer solutions are used through a spinneret comprising two co-axial capillaries (Figure 3d). One of the most common applications of the co-axial electrospun mats is in release of drugs by tailoring the core-shell composition of electrospun fibers. Thus, several studies can be found on the literature such as curcumin release [48], anticancer drugs release [49] or biocide release [50]. Summarizing, a large amount of diverse and smart material has been developed by using the electrospinning technique in any of its various setup configuration, giving place to advanced materials such as shape-memory electrospun mats [51], sandwich-type composite for packaging solutions [52] or reinforcement to epoxy resin films [53] among others.

In order to disperse NPs into the electrospun polymeric fibers as well as to improve the electrospun nanofibers performance, post-processing methods can be used (indirect approach) [54]. Among all the electrospinning post-processes, one of the most widely studied when working with NPs is the surface treatment of electrospun mats. In particular, surface treatment consists in the immersion of electrospun mats into a NPs solution [55]. Thus, the surface of electrospun fibers is covered by NPs thanks to electrostatic forces, hydrogen bonding or interactions among functional groups. Otherwise, NPs can be formed directly on the surface of nanofibers by in situ reduction. In this method, NPs are formed on the surface of electrospun nanofibers through a reducing agent. Electrospun mats are submerged into a precursor solution where a complex is formed between the metal ion and the functional group on the fibers, and once the mat is immersed, the NPs are synthesized by reducing the complex [56].

Sometimes, specific morphologies of NPs are required in the electrospun material. By a hydrothermal process, the formation and growth of crystals can be controlled. This method includes the various techniques of crystallizing substances from high temperature aqueous solutions at high vapor pressures. Basically, it is a way of synthesis of single crystals that depends on the solubility of minerals in hot water under high pressure [57]. In particular, many metal oxide NPs have been synthesized by this method (e.g., TiO_2_ [58], Al_2_O_3_ [59], Cu_2_O [60], etc.) for different applications as supercapacitors [61].

Unfortunately, nanoparticles commonly tend to aggregate, acting as defects. In this regard, gas-solid reaction is a well-known post-modification method that avoids this obstacle. By this method, electrospun fibers of polymer containing metallic precursor are prepared and then exposed to a special gas atmosphere. Nanosized particles are produced by exposing the surface of the nanofibers to the reactive gas. In most cases, surface modification leads to the loss of certain properties. In order to solve this problem, metal ions can be immobilized on the surface of nanofibers avoiding aggregation through a gas-solid reaction [62].

**Figure 4:**
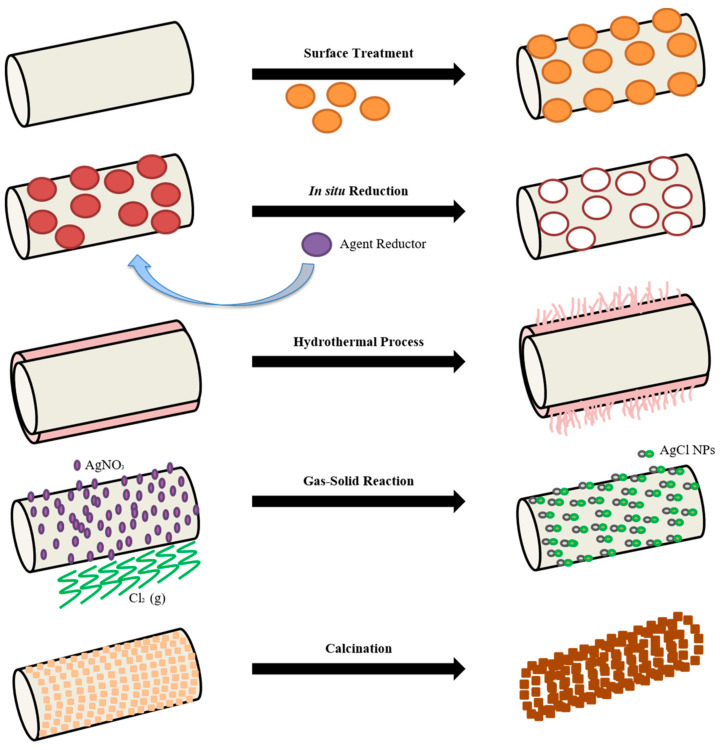
Schematic representation of post-electrospinning processes.

Furthermore, not always polymeric nanofibers obtained by directly electrospun polymers embedded with NPs are desired, since many applications need fully inorganic nanofibers where polymeric electrospun mats act as support for the obtaining of the inorganic array. In fact, many papers reported the fabrication of inorganic-based nanofibers by calcination process [63,64,65]. In this method, a blend of raw materials (polymer and NPs) is electrospun so that template nanofibers are obtained. Then, the polymeric matrix is calcined by heating under different atmospheres and completely inorganic oxide nanofibers are produced [66]. Therefore, many post-modifications can be applied after the electrospinning process. A brief summary of the most widely used ones are schematically represented in Figure 4.

Within the direct methods to obtain NPs-electrospun fibers, the most straightforward strategy is the direct fabrication, e.g., a stable suspension of NPs is added into de polymeric solution and then electrospun together in one step. The dispersion and agglomeration of NPs through the nanofiber can be controlled by varying the amount of NPs as well as the density of the solution. With this method, several electrospun nanocomposites reinforced with NPs have been produced for many different applications such as packaging technology [28,67,68] or tissue engineering [69,70].

## 3. Magnesium Nanoparticles (NPs) Role as Reinforcement of Nanofibers in Environmental Remediation, Energetic Devices and Industrial Catalysis Fields

Over the last few years, nanotechnology has been developing new materials and several methods to obtain organic-inorganic electrospun composites in nanometric scale. Among the inorganic elements, Mg-based NPs are becoming more and more studied in the last years, as reported before. Many different electrospun materials based on Mg compounds are being reported in literature, from magnesium oxide nanofibers obtained by calcination after the electrospinning process [71], to Mg-based NPs blended with polymer electrospun nanofibers with different morphologies [26,72,73,74]. The catalytic activity in chemical reactions with industrial interest, the thermal stability of Mg-based nanocomposites, and their biocompatible performance make them attractive candidates to be used in different fields, such as environmental applications, energetic devices, industrial catalysis, high temperature applications and biomedical field.

### 3.1. Environmental Applications

**Figure 5:**
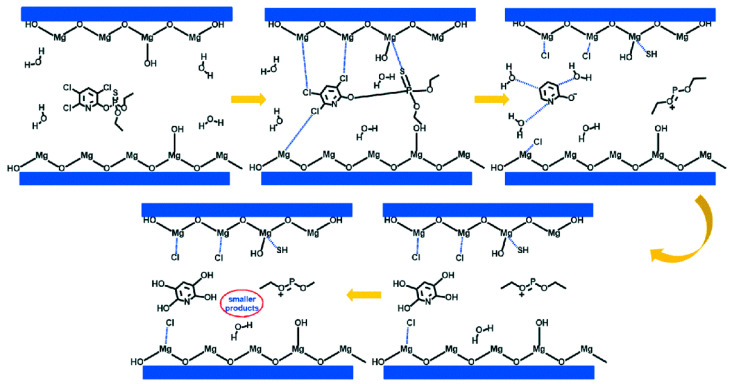
Plausible reaction mechanism for degradation of chlorpyrifos on the surface of MgO. Reproduced from [81] with permission from The Royal Society of Chemistry.

One of the most attractive applications of this kind of material is the removal of environmental pollutants from nature. Nowadays, the environment is becoming more contaminated due to large amounts of waste that finally end up in our ecosystems. The increase in pollution in recent years as well as the resulting health problems have led to the in-depth study of new ways of environmental remediation [75]. Among all the methods proposed to eliminate metal ions, organophosphates and dyes in residual waters, adsorption is the best in terms of feasibility and cost, and also the most widely used [76,77]. The Mg-based nanocomposites fabricated by electrospinning process are materials with ideal characteristics for being used in adsorption due to their large surface area. The Mg-based NPs adsorption performance has been reported only slightly in the literature. For example, Gao et al. [78] developed a method of controllable synthesis of MgO NPs with four different morphologies and found an excellent absorption performance for heavy metal ions and organic pollutants in water. In addition, some Mg-based NPs adsorption mechanisms of contaminant are also reported in literature. In particular, the mechanism of the adsorption process is based on electrostatic attraction ad surface complexation between the dye molecule and surface hydroxyl groups of the MgO adsorbent. Specifically, magnesium oxide NPs (MgO) was found to carry out oxidative degradation by cleavage of the P–S bond or P–O bond of organophosphates [79,80] and degradation of chlorpyrifos (organophosphate pesticide) by destructive chemisorption, reported in Figure 5 [81]. Thus, the researchers are focusing on the development of electrospun nanocomposites reinforced with MgO NPs for removal of toxic substances. 

Moreover, the use of a high active adsorptive such as MgO NPs in a network of nanofibers for removal of organophosphates provides some advantages over other materials. In 2014, Woo et al. [82] developed an MgO-embedded fiber-based substrate to be used as sorbent for toxic organophosphates. They prepared a polymer blend structure of cellulose acetate where MgO NPs were embedded, then an electrospinning solution of 60:40 acetronitrile-acetone and 15 wt% of 60:40 cellulose acetate-poly(ethylene oxide) (PEO) was used to obtain a electrospun mat with an average diameter of nanofibers of 1.35 ± 0.39 µm. In their conclusions, they reported the removal of 33% of methyl parathion from hexane solution in 100 min.

Additionally, the capacity of MgO NPs to degrade organophosphates has been tested in other works. Recently, Wei et al. [83] prepared a polyethersulfone nanofibrous membrane post-modified with dopamine and polyethylenimine reinforced with MgO NPs. They tested its degradation capacity against paraoxon-ethyl toxin, obtaining a 92% of removal in 40 min. Furthermore, Yu et al. [84] reported the synthesis of MgO mesoporous nanofibers and studied the adsorption capacity against fluoride and Congo red. The bumpy morphology of the fibers obtained by electrospinning process provided more active sites for adsorption, and the MgO nanofibers exhibited a surface area of 194.17 m^2^/g. This promising material showed an adsorption capacity of 237.49 mg/g for fluoride and 4802.27 mg/g for Congo red.

Furthermore, photocatalytic activity of electrospun MgO nanocomposite versus a widely used model reactive dye such as Reactive Yellow was reported in 2018 by Mantilaka et al. [85]. They fabricated the nanofibers using the electrospinning method via a polyvinyl alcohol (PVA)/magnesium precursor based system and compared its degradation activity with conventional MgO nanospheres. They reported a complete degradation of the reactive dye under ultraviolet (UV) irradiation for 100 min in the presence of photocatalytic MgO nanofibers. These results indicate that there are promising methods based on Mg for environmental remediation with good opportunity to be successfully used in the future. 

Another concern regarding environmental contamination is the presence of metal ions in water. Among them, the radioactive elements are considered the most toxic because once entered in vivo, they will cause serious damage to organs in the body. Thus, a suitable way of removing toxic metal ions from water is highly required. With this aim, Ren [86] reported the synthesis of MgO nanofibers and described its adsorption properties of radioactive metals. The nanofibers obtained could be used as adsorbents for the removal of uranium (VI). Specifically, the maximum adsorption reported was at pH 6, with an equilibrium adsorption amount about 90 mg/g achieved in 120 min. 

In this regard, heavy metals ions represent one of the major environmental concerns, as they show tendency to be accumulated into biosystems, which supposes a risk for public health. MgO NPs in combination with carbon nanofibers appear as a promising material for removal of heavy metal ions. Othman et al. [87] studied the adsorption of cadmium (Cd^2+^) ions by polyacrylonitrile-based carbon nanofibers using MgO as adsorbent. They demonstrated that the adsorption capacity of carbon nanofibers reinforced with MgO was higher when compared to the adsorption capacity of commercial granular activated carbon.

More recently, Almasian et al. [88] prepared mesoporous MgO/polypropylene glycol hybrid nanofibers in order to obtain a new adsorbent for the removal of heavy metal ions from solutions. They obtained mesoporous fibers with polypropylene glycol onto the surface with a specific surface area value of 185 m^2^/g. They tested the nanofibers against lead (Pb), cupper (Cu) and cadmium (Cd) and their results showed that the maximum adsorption capacity occurs at pH of 7.5. The regeneration experiment of nanofibers showed that the adsorption capacity for Cd, Cu and Pb was as high as 1900.05, 1919.28 and 1922.79 mg/g after seven cycles, respectively.

A recent study of Xu et al. [89] published in 2020, revealed a novel citric acid-assisted sol-gel method that did not contain any template or chemicals for the electrospinning of MgO-SiO_2_ ceramic fibers. The fibers obtained showed good morphologies with diameters of 1.23 ± 0.34 µm and were tested on lead (Pb), copper (Cu), methylene blue (MB) and fulvic acid (FA). The ceramic fibers showed high adsorption capacities of 753.1/481.0 mg/g for Pb/Cu and 315.6/24.0 mg/g for MB/FA. Later, they described the experimental data by Langmuir model and pseudo-second kinetic model for Pb and Cu adsorption [90].

Besides MgO NPs, magnesium hydroxide (Mg(OH)_2_) NPs are also being studied as environmental remediation material. In 2014, Jia et al. [91] followed an hydrothermal strategy to obtain polyamide 6 fibers with Mg(OH)_2_. The nanocomposite fibrous membrane obtained exhibited excellent chrome (Cr^2+^) removal performance with high performance and recycling property. The morphology of the nanofibers was found to be flower-like, which increases the specific surface area of the material. The promising results were a capacity of adsorption for chrome (Cr^2+^) of 296.4 mg/g of nanofibrous membrane and a removal percentage of 100% in the first cycle to 60% in the fourth cycle.

Other relevant materials studied in this field are layered double hydroxides (LDHs) which are nanostructured compounds containing positively charged brucite-like layers together with charge-balancing anions and water in interlayer regions. Based on this concept, Chen et al. [92] designed a novel adsorbent intercalating ethylenediaminetetraacetic acid (EDTA) into layered double hydroxides of aluminum and magnesium and subsequently encapsulated in a polymer matrix using electrospinning. The adsorption of the synthetized electrospun material was evaluated using Cu as target, and the maximum adsorption capacity reported was 120.77 mg/g, which reveals a great adsorbent with potential applications in water cleanup.

Beside the potential use of these materials in the cleaning of waste-water and removal of metal ions, they can also be applied to other fields. For example, Dehghan et al. [93] reported an appropriate electrospun nanocomposite of polyacrylonitrile (PAN) containing MgO NPs to be applied in air filtration. They optimized the electrospinning parameters and developed a filter medium comparable to high efficiency particulate air (HEPA) filter in efficiency and pressure drop. Later, they investigated the antibacterial properties of the produced nanofibers through disk diffusion and concluded that they can be usefully applied in air conditioning units to purify the air coming indoors as well as in local exhaust ventilation in industries or in fabric bag house [94]. However, the main electrospinning process conditions for obtaining the material reviewed above and their removal capability against environmental contaminants are summarized in Table 2.

### 3.2. Energetic Devices

The interest in new, affordable and sources of energy is one of the sustainable development goals and finding new materials that supply these needs is a subject of intensive research. The trend in recent years has been to develop miniaturized and low energy consumption equipment, for which the use of electrospinning technique has potential advantages to obtain functional materials that act as electrodes, due to its easy manipulation, good electrochemical performance and high aspect ratio.

Among the needs, there is a demand for miniaturization of rechargeable lithium ion batteries. In particular, LiCoO_2_ is the most attractive lithium battery material due to the high specific energy density and cycle life. However, LiCoO_2_ fibers studied previously, showed a rapid capacity loss because of the surface reaction of electrode materials with high surface areas [95]. In order to overcome this challenge, Gu et al. [96] studied the use of MgO as shell in LiCoO_2_-MgO fibers, obtained by co-axial electrospinning, to develop a promising cathode material for micro battery device technology. The parameters they used for the electrospinning process were the voltage at 25 kV while the distance between the spinneret and the collector was 30 cm. Thus, LiCoO_2_ co-axial fibers with diameter of 1–2 µm and MgO shell thicknesses of 50–100 nm were prepared. The electrochemical properties of the co-axial fibers were tested and the results showed that MgO coating avoid the spoilage of the surface. In addition, the cyclability was tested by charge–discharge experiment revealing retention of 90% in the discharge capacity after 40 cycles. In this way, in 2020, Ma et al. [97] studied how to improve the electrochemical performance of cathode materials for Li-S batteries using MgO. In this work, MgO and nitrogen-rich carbon nanofibers were prepared by the electrospinning process (15 kV) followed by post calcination treatment (900 °C for 3 h) and were used as cathode material in Li-S batteries. The resulting batteries exhibited high capacity, good cycle stability and rate performance, which delivered the initial discharge capacity of 846.75 mAh/g and 96.57% capacity retention after 100 cycles.

Other alternatives to MgO have been studied, for example spinel Co_3_O_4_ in different forms revealed reversible capacity, as well as ternary metal ions such as MgCo_2_O_4_. As it is widely known, the morphology of particles plays an important role in electrochemical performances and electrospinning is a very suitable method to obtain nanofibers with appropriate characteristics. With this aim, Darbar et al. [98] reported for the first time in 2016, the preparation of MgCo_2_O_4_ nanofibers and its electrochemical properties. The electrospinning technique was used with post calcination process at temperatures of 350, 550 and 750 °C. The average diameter of the calcined fibers was found to be 120–160 nm. The results showed that MgO acts as a buffer layer to prevent agglomeration of metal nanoparticles and improved conductivity. In addition, superior lithium storage for MgCo_2_O_4_ nanofibers was reported due to nanocrystalline fiber-type morphology and improved surface area.

Magnesium ferrite (MgFe_2_O_4_) nanocrystals have been explored as anode materials in lithium batteries with better theoretical capacities than carbonaceous anode materials [99,100]. The use of electrospun MgFe_2_O_4_ nanofibers as anode materials for lithium batteries was firstly reported by Qiao et al. [101]. They obtained electrospun fibers with an average diameter of 110 nm after calcination at 800 °C and described a reversible capacity of 409 mAh/g after 100 cycles. 

Additionally, the search for advanced materials that innovate in the energy industry led to the development of novel technical textiles, such as photovoltaic and photocatalytic textiles. These kinds of textile are formed by photovoltaic cells onto fabric substrates so that the energy of light is converted into electricity. In particular, dye-sensitized solar cells (DSSCs), usually made of TiO_2_ NPs, act as photoanodes. In order to increase the DSSCs, some papers reported the covering of TiO_2_ NPs with metal oxides [102]. Thanks to the wide bandgap, MgO NPs used as coating layer is able to improve the efficiency of DSSCs due to the improvement of dye adsorption. In this sense, Du et al. [103] reported the synthesis of co-electrospun TiO_2_/MgO nanorods by electrospinning and post calcination indicating its application in photocatalytic functional textile. 

**Figure 6:**
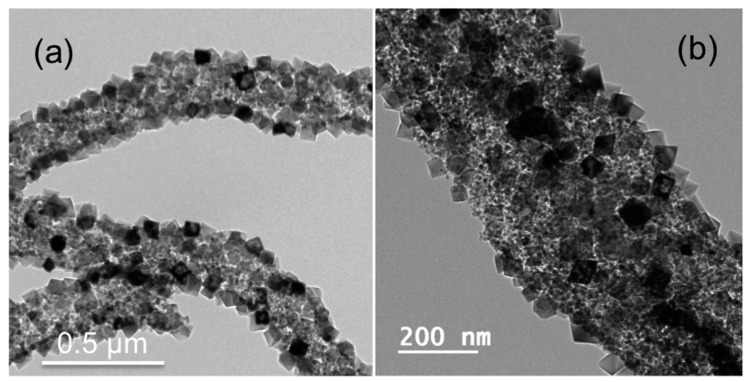
(**a**) Bright field transmission electron microscope (TEM) image of MgO nanoparticle (NP) decorated MgO fibers prepared using a solvent mixture of (N,N-dimethylformamide (DMF) + methanol). (**b**) TEM image at high magnification showing the uniform distribution of octahedron shaped MgO NPs decorated on MgO nano fibers. Reproduced from [104] with permission from The Royal Society of Chemistry.

They prepared two novel textiles using the TiO_2_/MgO nanorods, one was flexible DSSCs incorporating the synthetized material, and the other was photocatalytic functional textile. Both of them exhibited better efficiency and activity than their counterparts based on pure TiO_2_. They proposed the formation of an energetic barrier at the interface between TiO_2_ core and MgO shell that would probably avoid the undesired charge recombination. More recently, in 2017, Sainudeen et al. [104] reported the synthesis of hollow MgO nanofibers reinforced with MgO NPs where they combined the large surface area of NPs and the anisotropic properties of hollow nanofibers. This combination reveals a potential material to be used in DSSCs and batteries. The nanocomposite was prepared using electrospinning and thermal treatment post-processing, using a solution containing a polymer and a MgO precursor with cetyl trimethyl ammonium bromide (CTAB) as surfactant. Different morphologies of NPs were observed depending on the concentration of CTAB in the solution used, finally they controlled the size distribution and the shape of NPs (Figure 6).

MgO has been also chosen as dopant metal oxide to produce ZnO alloys with higher band gap for possible quantum well structures [105,106]. It is widely reported the enhancement of the band gap of the ZnO by using Mg as dopant [107]. Different methods have been used to produce ZnO nanomaterials. Cetin et al. [108], chose electrospinning technique and post calcination in air atmosphere. In their studies, they prepared undoped and Mg-doped ZnO nanofibers with different doping concentrations. They calcined the nanofibers at 300, 400, 500 and 600 °C and characterized the electrospun material. The study of the electronic band transition in as-deposited and calcined films revealed that the exitonic transition energy of the ZnO nanostructure blue-shifted to a high energy value with an increasing Mg doping ratio. The emission spectra of the samples determined the decomposition temperature of the electrospun as 320 °C. In addition, Orujalipoor et al. [109] studied the preparation of films with MgO doped ZnO nanocrystalline ceramics on Si commercial glass wafers and characterized the morphological changes by small and wide angle X-ray scattering methods. In this case, the electrospinning process and post-calcination was carried out at 320, 340, 360 and 380 °C. The in situ growth of nanostructures was investigated and the results showed that Si is more convenient as substrate to create periodic lamellar and big size nanoformations in the surface of the films, while the high uniformity of the deposited films were obtained for 360 °C. The presence of MgO as dopant played an important role in the primary growth stage acting as seeds for the generation of doped nanocrystals with different shapes.

As previously reviewed, Mg-based compounds are widely used in electrospinning research focused on energy fields. With the advance of industry, many scientists are seeking new strategies, which included many fields of knowledge, to solve problems such as new energy storage systems, development of electronic devices or the fabrication of novel sensors. In this way, the electrospinning technique has revealed as a versatile tool that is the focus of many studies that try to remedy the energy and industrial needs that we find today. For example, Choi et al. [110] worked in field effect transistors (FET), using high-mobility one-dimensional semiconducting nanofibers to achieve more reliable FET devices and higher device yields.

Other researchers are focusing their work on the development of electron emitting devices which can be used in field emission displays or scanning electron microscopes. Despite the attention given to carbon based materials, their emission current stabilities are still low. With the aim of improving field electron emission, the use of doping materials with wide band gap such as MgO, is gaining interest. Electrospinning process allows a direct method to obtain carbon nanofibers reinforced with metal oxides in order to fabricate promising materials for electron emitting devices. For example, Aykut [111] reported the synthesis of MgO-reinforced electrospun carbon nanofibers prepared by electrospinning and post calcination. The results showed that the addition of MgO displayed enhanced field electron emission as compared to that of pure carbon nanofibers. 

The development of efficient energy storage devices has recently been explored in order to satisfy the society energy demands. In this field, supercapacitors are considered the main energy storage systems because of their long life-cycles and rapid charging-discharging capacity. Carbon nanofibers are widely used as electrode materials in supercapacitors but, in order to improve the performance of carbon-based materials, some investigations have been carried out. One of them consists in doping with nitrogen to enhance the capacity and obtain superior cycling stability. In their study, Tan et al. [112] used Mg(OH)_2_ in the electrospinning process as starting materials and then used calcination to obtain magnesium oxide. They proposed MgO as a hard template to prepare porous carbon fibers with N-doping due to its low cost and easy removal. In addition, they used a synthesis process of the Mg(OH)_2_ nanoplates because of its low cost and for being environmentally friendly.

### 3.3. Industrial Catalysis

Beside the previously reviewed applications, nanomaterials are receiving continue attention in the literature for being effective catalyst against chemical warfare agents [113,114]. In particular, the capacity of MgO to adsorb chemical and biological warfare agent has been widely reported [115,116,117] and, furthermore, the degradation products derived from the catalytic activity have been found to be non-toxic. The combination of electrospun polymer with MgO NPs in order to obtain new full protecting materials is becoming a regular topic in safety research, protecting cloth development and full barrier protection. For example, Sundarrajan et al. [118] fabricated a nanocomposite membrane of poly (vinyl chloride) (PVC), poly(vinylidene fluoride-co-hexafluoropropylene) (PVDF) and polysulfone (PSU) nanofibers reinforced with MgO NPs. The catalytic activity was tested against paraoxon, a class of organic chemical that disrupt the mechanisms by which nerves transfer messages to organs. The reactivity of the resulting membranes was found to depend on the polymer used, probably due to the reactivity of the NPs with functional groups in polymer chains. The fabricated nanocomposite membrane (containing 5 wt% MgO) was found to be about 2 times more reactive than the currently used charcoal, being 35% the maximum nanofilling percentage of MgO that could be electrospun. In the same way, Ryu et al. [119] demonstrated the activity against chemical warfare agents of nanofibrous nanocomposites impregnated with MgO and polyoxometalate. They prepared a layered material consisting in an outer layer of nanofibers and amphiphobic absorbent, so that the liquid chemical warfare agent will bounce, and an inner layer composed of Nylon 6,6 nanofibers with MgO and polyoxometalate that act absorbing gas chemical warfare agents. The results showed that the outer layers were not wetted by water and other chemical warfare agents (2-chloroethyl ethyl sulfide and dimethyl methylphosphonate). However, the inner layers were found to be inadequate to be used singly. Furthermore, the properties of the assemblies could be controlled by the number of layers used. As a result, the material could provide good protection while still allowing water vapor transmission. 

Nanofibrous mats reinforced with MgO NPs have been not only proposed as future protective clothes against chemical warfare agents but also as future textiles for daily use, for example as UV-protection. Usually, the normal clothing is inadequate to protect the skin from UV radiation damage [120], so new protector agents are being investigated in order to increase their effectiveness against UV radiation. Thanks to the potential in UV radiation absorption of metal oxide nanoparticles [121,122] and their large specific surface area, mixing MgO NPs and electrospun polymers by electrospinning appears as a promising alternative for high-performance textiles. For example, Dadvar et al. [123] prepared and described the UV-protection properties of electrospun polyacrylonitrile nanofibers reinforced with MgO and Al_2_O_3_. The addition of MgO and Al_2_O_3_ NPs in the electrospun mat increased its UV-protection capacity considerably. The mats obtained with area density of 5.64 g/m^2^ containing 10 and 15%wt MgO were classified as very good UV-protection layers according to the Australian/New Zealand Standard AS/NZS 4399 (1996).

It has been well documented that wavelengths in the ultraviolet radiation range of the solar spectrum can be absorbed by skin and lead to cutaneous injury and various other deleterious effects. In particular, UV radiation can delay wound-healing progress due to the inhibition of keratinocyte motility [124]. At this point, UV radiation absorption of Mg-based NPs has to be considered as an additional property in the development of new materials for biomedical applications.

In addition, MgO is an organic ceramic material of great importance and has received a great deal of interest due to its potential chemical and electronic applications for possessing unique magnetic [125], optical [126] or thermal [127] properties. Due to its high reactivity and versatility, MgO has been selected as one of the most promising catalyst supports. One of the most attractive properties of Mg-based NPs is their large surface area previously described. Metal oxide nanoparticles have been described as good candidates to enhance the adsorption capability of electrospun polymers. These materials can be used in a wide variety of applications in which one of the most important is the storage of methane. Clean and efficient energy such as natural gas are attracting much attention, however the handling and storage of this liquefied gas results in being challenging [128].

To overcome these problems, scientists are focusing on absorbed natural gas as an alternative way of storage [129]. For this purpose, large surface area materials are required with excellent capacity of adsorption. In this sense, electrospun polymers reinforced with NPs are suitable candidates. Some recent publications demonstrate the ability of MgO NPs to improve the adsorption capacity of electrospun fibers. For example, activated carbon nanofibers (ACNF) embedded with MgO and MnO_2_ were fabricated and studied by Che Othman et al. [130]. The results showed a surface area of up to 1893 m^2^/g for ACNF/MgO. These results are supported by the fact that the incorporation of metal oxides helps to activate the ACNF by catalyzing the process. In addition, the volumetric adsorption test revealed a CH_4_ uptake of 2.39 mmol/g for ACNF/MgO, the highest in comparison with the rest of electrospun materials studied. It is worth noting that ACNF modified with MgO have a great impact on the CH_4_ storage capacity due to the differences in the specific surface area and pore volumes. Specifically, in 2019, Ma et al. [131] demonstrated for the first time, the synergistic promotion effect of MgO and CeO_2_ on the Ni/Al_2_O_3_ catalysts for methane partial oxidation through optimizing MgO and CeO_2_ contents in the MgO-CeO_2_-Ni/Al_2_O_3_ catalyst system. They prepared nanocomposites with different content in MgO by electrospinning process and post calcination in air at 800 °C for 1 h. They observed how MgO influenced catalyst performance to different extents. As 5 wt% of MgO was added, CH_4_ conversion was increased from 5% to 12% for the MgO-Ni/Al_2_O_3_ catalyst and from 11% to 85% for the MgO-CeO_2_-Ni/Al_2_O_3_ catalyst. Therefore, the synergistic effect of MgO and CeO_2_ contributed to the high catalytic activity. Furthermore, MgO has been proposed as a catalyst to improve the hydrogen storage performance of carbon nanofibers. They prepared magnesium oxide-carbon fibers nanocomposites by electrospinning of polyvinylpyrrolidone and MgO NPs at 18 kV of voltage. The rotating drum collector was placed at 15 cm from the tip and the solution was pumped at 1.5 mL/h. Finally, a post calcination process was carried out at 800 °C for 2h. The addition of MgO increased the specific surface area of carbon nanofibers, which provided more hydrogen adsorption sites and subsequently improved the hydrogen storage capacity in a 30% of capacity at room temperature compared with neat carbon nanofibers [132]. More recently, in 2020, Triviño et al. [133] studied the CO_2_ sorption capacities of an eutectic mixture of KNO_3_ and LiNO_3_ in an MgO fiber matrix via core-shell electrospinning. The MgO fibers acted as a protective shell that prevents structural changes and allowing the sorbent to retain its cyclic stability after multiple cycles. 

As can be appreciated, the current trend is to develop inorganic-organic nanocomposites to gradually satisfy the challenges that the industry generates. The addition of NPs enhances the performance of some promising materials that are widely studied, such as ACNF, and enlarge their application fields. Taking advantage of the large specific surface area of ACNF reinforced with NPs, the catalytic activity of these materials has been tested with some chemical reactions of industrial interest. For example, Liu et al. [134] studied the catalytic activity of carbon nanofibers decorated with MgO-Ag NPs or Al_2_O_3_-Ag NPs in the epoxidation of styrene, obtaining conversion yield of 43% and 46.45%, respectively. As can be seen, the use of MgO NPs as a kind of catalyst not only reduced the amount of catalyst but also improved the efficiency of catalytic reaction. 

### 3.4. High-Temperature Applications 

Finally, another important characteristic of MgO nanofibers is their high melting point (2850 °C) and low thermal conductivity [135] that makes MgO an attractive material to be used in high-temperatures applications. With this aim, Xu et al. [136] reported a possible formation mechanism of MgO nanofibers obtained by electrospinning process through mixed precursor (magnesium acetate and magnesium citrate). The fibers with magnesium citrate, mass content in the range of 30–50%, exhibited good high temperature stability and the original feature was preserved after being heat-treated at 1000 °C. The decomposition process of the fibers is relaxed due to the fact that the mixed precursors extended the crystallization temperature range.

## 4. Development of Electrospun Polymers Reinforced with Mg-Based NPs in Biomedical Applications

The tissue-engineering field of research incorporates cell biology, basic medical sciences, biomaterials and biomedical engineering [137,138]. Its main purpose is to produce biological substitutes that restore, maintain, or improve tissue function by different strategies. One of the strategies is to fabricate biocompatible scaffolds that would support, or ideally enhance, regeneration of tissue within the injured area after biomaterial implantation. A scaffold should also guide the growth of cells and their organization in three dimensions [139] and serve as a temporary support for cell attachment and differentiation [140]. 

The aim of the engineering biomaterials is to fabricate a biocompatible scaffold that would support or ideally enhance regeneration of tissue after biomaterial implantation within the injured area. Living human tissues are a complex system with special biochemical and biomechanical properties so that the artificial substitutes have to successfully mimic the natural chemical and biological environment in the human body. Thus, biocompatibility, porosity and mechanical properties are the features that ideal biomaterials have to show together with others specific requirements in order to be suitable as bone tissue-engineering substitutes. These requirements are summarized in Table 3.

Biocompatible scaffolds have to support normal cellular activity (cells attachment, migration, proliferation, differentiation) without any toxic effects to the host tissue [148]. Several research projects have been carried out about the biocompatibility of both natural and synthetic electrospun polymers [149,150,151]. Among the first, chitosan [152], collagen [153], gelatin [154] or cellulose [155] have been widely explored in tissue engineering due to their bio-based origin, renewability, biocompatibility and biodegradability. Synthetic polymers such as poly (ε-caprolactone) (PCL) or PVA are among the most studied especially because they are easy to electrospun and present good mechanical properties [156,157]. In addition, electrospun fibers obtained by blending natural and synthetic polymers are also studied, thus synergistically bringing together their biological function as well as their mechanical response. In particular, in Table 4 the main characteristics and the immunological profiles of both synthetic and natural polymers are summarized, as previously reported by Mariani et al. [158]. 

Suitable porosity is other essential requirement of scaffolds to mimic the natural extracellular matrix. Indeed, a proper porous structure for bone and cartilage regeneration should support cell growth, present good surgical handiness and at the same time show mechanical properties adjusted to the implantation area (mean mechanical properties of living tissues and some available commercial materials are summarized in Table 5). More specifically, the mechanical properties should be similar to those of native tissue for proper load transfer in order to avoid stress shielding that would cause excessive bone resorption and implant loosening [159,160]. 

To achieve biomaterials with mechanical properties close to living tissues, research mainly studied polymeric nanocomposites where the polymeric matrix mimics the flexible organic part of the tissue and nanoparticles provide better mechanical properties of the resultant scaffold [161]. Electrospun polymers reinforced with NPs have been widely investigated due to their high surface area which allows the diffusion of nutrients and oxygen for cell survival. The electrospinning technique allows fabricating woven no-woven electrospun nanofiber mats with controlled fiber diameter and unique architecture which can recreate the natural human environment. 

In this way, NPs based on magnesium have been recently studied as reinforcement due to the importance of this material into the human body. Mg intracellular cations act as cofactor of enzymatic reactions, and are essential for the synthesis of proteins and nucleic acids [162,163] and for the transport of potassium and calcium ions [164].

**Table 5:** Summary of mechanical properties of living tissues and commercial biocomposite materials.

**Living Tissue**	**Young Modulus (MPa)**	**Ultimate Tensile Strength (MPa)**	**Elongation At Break (%)**	**Ref.**
Aorta valve, human	2–15	0.4–2.6	0.22–0.30	[165,166]
Mitral valve anterior leaflet	3.6 ± 1.8	0.05–0.45 Anterior 0.10–0.20 Posterior	-	[167,168]
Mitral valve chordae tendinese, human	330 ± 228 to 388 ± 290	36.8 ± 22.5 to 40.8 ± 24.6	0.20 ± 0.09 to 0.21 ± 0.12	[169]
Skin, human	3–54	1–20	30–70	[170]
**Wound Dressing Material**	**Young Modulus (MPa)**	**Ultimate Tensile Strength (MPa)**	**Elongation At Break (%)**	**Ref.**
Omiderm	60.4 ± 4.5	-	56.3 ± 3.0	[171]
Chitosan-Alginate + Alphasan^®^ + Silpuran^®^	-	43.5 ± 5.5	3.9 ± 0.5	[172]
**Dental Membranes**	**Young Modulus (MPa)**	**Ultimate Tensile Strength (MPa)**	**Elongation At Break (%)**	**Ref.**
Bio-Guide^®^	15.7	4.8	-	[173]
Collprotect^®^	158.5	13.1	-	[173]
Jason^®^	178.9	13.0	-	[173]

According to the role of magnesium in cellular functions, various types of synthetic and bio-polymers reinforced with Mg-based NPs have been reported in the last few years as promising candidates for bone replacement therapies due to the stimulation capacity of bone cell differentiation in vitro [174] and bone formation in vivo [175]. These new materials are focused on bone implants and bone growth applications due to the crucial role that magnesium plays in bone development. About 50–60% of the total body Mg^2+^ content is kept in bone. Serum Mg^2+^ concentrations are closely related to bone metabolism; bone surface Mg^2+^ is continuously exchanged with blood Mg^2+^. In bone, hydroxyapatite structures mainly consist of P and Ca^2+^ and are bound by Mg^2+^ ions at the surface of the hydroxyapatite crystals. Mg^2+^ increases the solubility of the minerals and thereby plays an important role on the hydroxyapatite crystal size [176]. Consequently, Mg^2+^ deficiency results in decreased bone formation.

**Figure 7:**
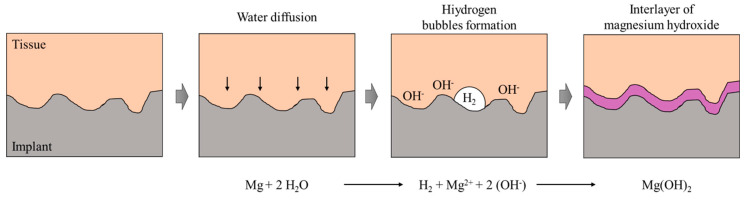
Schematic representation of Mg corrosion reaction with water into the human body.

However, when Mg-based NPs are used as reinforcement of polymers in biomedical implants, the reaction between the biological environment of the surrounding tissue and magnesium has to be taken into account, since water molecules react with Mg in a corrosion reaction resulting in hydroxide ions (OH^−^) and hydrogen gas (H_2_). The problem with hydrogen production is the capacity of the human body to metabolize the gas generated in high concentrations which could be accumulated at the implant area spoiling the growth of new tissue. In addition, the hydrogen bubbles can reach the blood circulatory system resulting in the patient’s death. The hydroxyl groups generated in the reaction also increase the pH and react with Mg^2+^ ions causing the precipitation of Mg(OH)_2_. Thus, a passive interlayer of magnesium hydroxide is formed on the degradation surface [177] (see schematic representation in Figure 7). The rapid corrosion, generation of a large volume of hydrogen gas, accumulation of the hydrogen bubbles in the region adjacent to the implant, and increase in local pH value of body fluid, are the most critical limitations in using Mg-based NPs in medical implants. Hence, controlling the corrosion rate of Mg inside the human body is an important issue to address in development of magnesium based biodegradables implants [178].

In order to improve and control the high degradation rate of magnesium under physiological conditions, some researches have been carried out during the last years. Several strategies have been developed such as the use of Mg particles surrounded by a biodegradable and biocompatible polymer as PLA [179,180]. To obtain a strong particle/matrix bond, Ferrández-Montero et al. [181] modified the Mg particles surface to provoke a stable suspension to be used in the processing of films prepared by tape casting with different content in magnesium. In this way, they obtained Mg microparticles embedded homogenously in the polymer matrix and protected by a PLA layer. The incorporation of Mg microparticles produced an improvement in PLA hydrophilicity and decreased the gas permeability. The hydrogen release rate of these materials was found to be under the maximum tolerable hydrogen release for the human body (2.25 mL/cm^2^/day) and the cell viability assay proved that the presence of Mg^2+^ ions promoted the proliferation of mouse embryotic fibroblast cells and compensated the pH decrease associated to PLA degradation. 

For all the above, it is clear that it is very important to take into account on the one hand the requirements needed to achieve a suitable biomedical device and on the other one, the important role of Mg in human metabolism and the challenges of using this element as nanofillers for biodegradable implants. Moreover, thus considering that electrospinning is a suitable processing technique for tissue engineering, therefore, in the next paragraph will be summarized the main effort for electrospun polymers with Mg-based NPs published in the biomedical area.

Asgharnia et al. [182] reported in 2013 the synthesis and characterization of SiO_2_-CaO-P_2_O_5_-MgO based bioactive glass and glass-ceramic nanofibers for biomedical applications. They used poly(vinyl pyrolidone) to obtain electrospun nanofibers from 246 nm to 156 nm after calcination at 600 °C. They tested the bioactivity of the material in vitro in simulated body fluid solution showing that the prepared nanofibers have rather good biomineralization properties, so that after immersion for 12 h, calcium phosphate nanoparticles were formed and covered the surface of the nanofibers.

The tendency to use polymers that are biodegradable and tolerable by the human body has also been reflected in research with Mg-based NPs. Boakye et al. [183] fabricated and characterized electrospun poly (ε-caprolactone)-MgO-keratin-based nanofibers. They chose keratin for being a natural polymer, found largely in hair and fingernails and for its biological activity as a cellular anchor. They obtained a fiber diameter average of 0.45 µm and the addition of MgO slightly increased the mechanical properties of the material in comparison with neat polymer nanofibers. Overall, the nanofibers reinforced with keratin and MgO (ratio 1:1) releases more Mg^2+^ over time compared to the other nanofiber ratios studied.

PCL-based nanocomposite is one of the most investigated in the biomedical field. For instance, Souza et al. [184] developed a new biocomposite based on bioactive glass microfibers (SiO_2_-Na_2_O-K_2_O-MgO-CaO-P_2_O_5_) in membranes of PCL nanofibers for potential nerve guide application. The presence of bioactive glass microfibers increased the mechanical properties of the material so that the tensile strength was tripled (60 ± 16 MPa) with no effect on the nerve guide flexibility. Moreover, the permeability test revealed that the developed biocomposite was permeable to water vapor, a crucial skill for nerve guide since it permits the exchange of growth factors and excretions between the nerve guide and the medium.

Other examples of biodegradable natural polymers reinforced with MgO NPs can be found in the literature. De Silva et al. [185] published in 2017 results on an alginate-based nanofibrous scaffold reinforced with MgO NPs. They used near-spherical shape NPs with an average diameter of 45 nm, obtaining alginate/MgO nanofibers with a diameter ranging from 60 to 250 nm. The mechanical properties of the material were found to be improved with the addition of MgO NPs. The tensile strength and the elastic modulus of alginate/MgO 10% (w/w) were the highest among the samples studied while retaining the inter fiber porosity. These results suggest that the proposed material could be a suitable candidate to be used as an artificial substitute for extracellular matrix in biomedical applications. Similarly, Suryavanshi et al. [186] fabricated a nanocomposite electrospun fiber scaffold of PCL reinforced with MgO NPs and reported the in vitro and in vivo evaluation. Firstly, they synthetized the MgO NPs by hydroxide precipitation sol-gel method, obtaining a size range of 40–60 nm and selected 10% (w/w) of MgO NPs. Then, the electrospinning conditions were set as follows: feed rate = 1.9 mL/h, voltage = 19 kV and solvent = trifluoroethanol, obtaining a diameter of fibers ranging from 200 nm to 600 nm. The uniform distribution of MgO NPs improved the tensile properties (tensile strength: fourfold, and modulus: threefold) and showed a great performance in vitro with normal tissue response after implant in Sprague Dawley rats.

**Figure 8:**
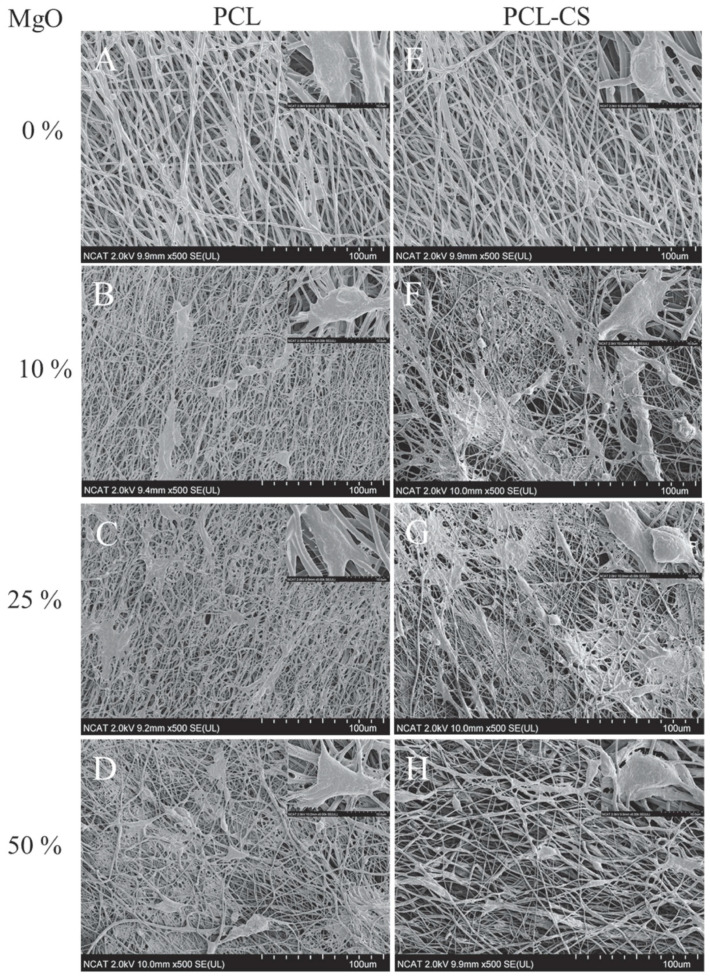
Scanning electron microscope (SEM) images showing the morphology of 3T3 fibroblast cells seeded on nanofiber membranes for 3 days. Images (**A**–**H**) represent poly (ε-caprolactone) (PCL), PCL/MgO (90/10), PCL/MgO (75/25), PCL/MgO (50/50), PCL/CS, PCL-CS/MgO (90/10), PCL-CS/MgO (75/25) and PCL-CS/MgO (50/50) respectively. Insets are the higher magnification images of the corresponding SEM images of the nanofiber, reprint form reference 187.

More recently, Rijal et al. [187] reported a new advance in the development of nanocomposite electrospun materials. They prepared nanofibers of MgO, PCL and Chitosan (CS) by electrospinning process, setting the processing conditions as follows: flow rate = 2.5 mL/h, voltage = 25–27 kV and solvent = trifluoroethanol. With this setup, nanofibers with diameters in the range of 0.7–1.3 µm were fabricated with different ratios of MgO NPs, as indicated in Figure 8.

They reported that PCL/MgO showed the highest Young modulus (~25 MPa) compared to other compositions studied but the highest ultimate tensile strength was obtained with PCL/CS nanofibers (~3 MPa). The proposed material showed no toxicity and cell proliferation with a viability >75%, which is considered a safe level.

In order to develop new materials to be used during the bone regeneration process, Lee et al. [188] prepared novel oriented bioactive glass/PLA scaffolds by controlling cell alignment and proliferation, which play important roles for achieving bone anisotropy and bone mass, respectively. They studied electrospun fibers mats containing bioactive glasses and blends of bioactive glass with CaO, MgO or SrO in different percentages. They reported that oriented electrospun fiber mats enabled cell alignment along the fibers and promoted cell proliferation due to the ions released from the bioactive glasses. The cell proliferation was significantly regulated by the releases of Mg^2+^ and Sr^2+^ and the osteoblast proliferation was improved. 

Electrospun nanocomposite scaffolds based on polyurethane have also been tested in this field. Thus, Mani et al. [189] investigated polyurethane reinforced with MgO NPs and neem oil for regenerative medicine. The addition of MgO NPs reduced fibers diameter from 1000 ± 176.74 nm for pristine polyurethane to 522 ± 159.10 nm for the highest amount of MgO NPs. Also, the crystallinity behavior of the electrospun materials was altered by the addition of MgO NPs and neem oil. Regarding the mechanical properties, the addition of nanofillers increased the tensile strength from 6.63 MPa (pristine polyurethane) up to 10.15 MPa (4 wt% MgO NPs).

Other electrospun materials reinforced with Mg(OH)_2_ NPs have been reported for biomedical applications. Romeo et al. [190] encapsulated inorganic double hydroxide (Mg-Al) into PCL. The structure of layered double hydroxides (LDHs) simulates the original packaging of layers in brucite. In this mineral Mg atoms show octahedral coordination where each atom is surrounding by OH groups. LDHs show a great ability to undertake ion-exchange procedures which makes this material a potential host of active molecules with controlled release. They studied a completely inorganic LDH carbonate and one organically modified with 12-hydroxydodecanoic acid (LDH-HA). The electrospinning process produced PCL and PCL/LDH nanofibers with an average diameter of 600 ± 50 nm whereas PCL/LDH-HA showed an average diameter of 300 ± 50 nm. A global brief electrospinning processing conditions, mechanical properties obtained and potential applications of the researches reviewed above are summarized in Table 6.

**Figure 9:**
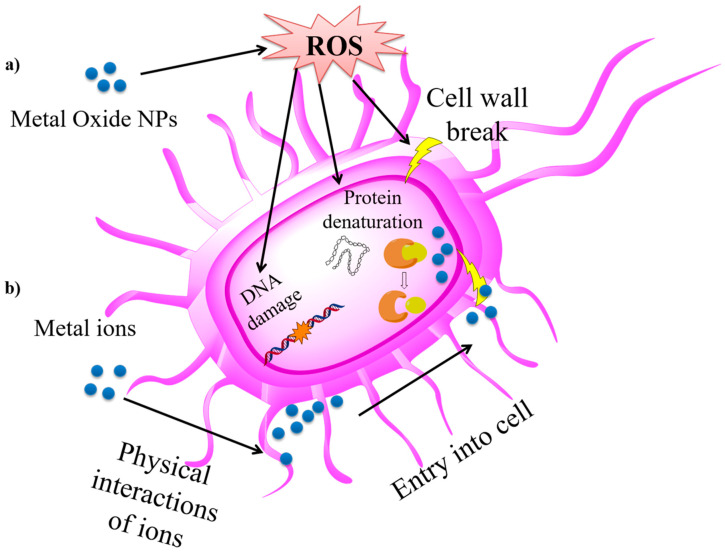
Schematic representation of antibacterial activity of metal oxide NPs adapted from reference [192]. (**a**) By generation of reactive oxygen species (ROS). (**b**) By generation of metal ions.

In addition, over the last few years, several studies have suggested that metal NPs are excellent antibacterial agents [191]. Many papers reported antibacterial activity of metal oxides NPs presenting no toxicity towards humans at concentrations used in the electrospinning process [192]. Focusing on Mg-based NPs’ antibacterial activity and according to the reports, MgO NPs damage the cell membrane causing the leakage of intracellular contents which in turn leads to the death of bacterial cells [193]. Several studies have been carried out to elucidate the antibacterial mechanism but they are still not clear. Two main mechanisms are described: on the one hand, the NPs could generate reactive oxygen species (ROS) that provokes bacterial cell death, and on the other hand, metal oxide NPs could generate metal ions that interact with bacterial cells [194,195,196]. A schematic representation of these mechanisms of bacterial death is shown in Figure 9.

In recent years [197,198], thanks to the fact that MgO NPs show bactericidal activity against both Gram-positive and Gram-negative bacteria [199], together with their ability to improve heat resistance and their potential fire-retardant property, they have been investigated as suitable materials to be used as inorganic reinforcement for electrospun nanocomposite mats. For example, Venkatram et al. [200] described an Ag-MgO/Nylon 6 electrospun nanocomposite for protective applications. They obtained bead-free nanofibers with average diameter of 35 nm and 55 nm when MgO (3 wt%) and AgNO_3_ (0.5 wt%) have been added, respectively. While the Nylon 6 + MgO (3 wt%) showed a reduction in the number of colonies of 41% against *Escherichia coli* and 21% against *Staphylococcus aureus*, by combining MgO and AgNO_3_ NPs in different ratios a decrease of 88% and 54% was achieved against *E. coli* and *S. aureus*, respectively. Furthermore, the combination of MgO and AgNO_3_ not only increased the antibacterial activity, but also provided good flame retardancy (burning rate of 1.56 mm/s). In this regard, Mg-based NPs are also used to develop promising fireproof clothes. For example, Zheng et al. [201] reported the synthesis of nanofibers coated with Mg(OH)_2_ NPs by wet electrospinning. They placed the flame-retardant NPs exclusively on the surface of the fibers in order to impact its combustion behavior. The results show that nanofibers with NPs on the surface were fire resistant while nanofibers with NPs inside burned rapidly upon exposure to an open flame. However, another example of antibacterial use of Mg-based materials is reported recently by Bakhsheshi-Rad et al. [202], who deposited Ta_2_O_5_ compact layer and PCL/MgO-Ag nanofibers porous layers on Mg alloys to improve anticorrosion and antibacterial performance of orthopedic implants. The electrospun nanofibers coated alloy show greater corrosion resistance than Ta_2_O_5_ coated alloy or uncoated specimens. Also, the nanofibers show enhance antibacterial behavior toward Gram-negative (*Escherichia coli*) and Gram-positive (*Staphylococcus aureus*) than Ta_2_O_5_ coated alloy and uncoated specimens. Furthermore, piezoelectric materials play an important role in this field. For instance, Hussein et al. [203] reported in 2019 the production of biocomposites from polyvinylidene fluoride with the addition of MgO NPs by electrospinning. They reported that the inhibitory zones against *Escherichia coli* and *Staphylococcus aureus* increased as the weight fraction of MgO NPs increased, with 7 wt% MgO NPs being the amount that obtained the maximum inhibition zone for both bacteria. Additionally, the Fourier transform infrared spectroscopy studies revealed the presence of piezoelectric β-phase at 840 cm^−1^ wave into the electrospun biocomposites, which is necessary for advanced biosensors used in sensing wound healing.

As can be appreciated, the use of MgO NPs and Mg-based compounds in tissue engineering has become a promising option for researches in medical field in order to mimic the mechanical properties of living human tissues (Table 3 and Table 5). The addition of Mg-based inorganic nanoparticles can improve the mechanical properties of electrospun materials as well as the cell proliferation. Although MgO NPs present several advantages against pathogenic bacteria, it can be considered challenging using them as antimicrobial agents thus taking into account that many efforts are need for studying their health effects on cells, tissues and organs. It is known that the in vivo performance of nanoparticles relies heavily on its ability to properly interact with biological systems [204]. Once nanoparticles are present in the physiological medium, plasma proteins will be rapidly adsorbed onto the nanoparticle surface, which can not only deteriorate targeting capability, but also dramatically increase immune clearance [205]. However, some strategies are being studied in order to enhance the nanoparticles’ performance inside the human body such as shielding nanoparticles with polyethylene glycol (PEG) for reducing unwanted biological interactions [206] or increasing the specific interactions between nanoparticles and its desired target [207]. In order to overtake this problem, the future use of pure Mg NPs has to be taken into account due to the recognized role that Mg plays in bone formation and regulation of calcium homeostasis that supposes a healthy inorganic reinforcement into polymeric composites. Moreover, the trend nowadays is to fabricate electrospun mats reinforced with MgO NPs by direct electrospinning as it summarized in Table 6, representing a significant advance in the development of nanocomposite materials with structural and biological properties that will be useful for biomedical applications.

## 5. Conclusions

The potential applications of magnesium-based polymeric nanocomposites obtained by electrospinning technique have been reported in this review. It is true that there are several reviews that describe the processing, use and characterization of electrospun nanocomposites, however, based on our knowledge, no review on electrospun nanocomposites reinforced with nanoparticles based on magnesium, Mg-based NPs, are reported still now. First of all, the importance of using electrospinning techniques has been considered, focusing the attention on the optimization of the processing-window as well as the main results reported up to now, in term of electrospun polymeric fibers reinforced with Mg-based nanoparticles obtained by direct electrospinning process or by post-processing treatments. In fact, even if electrospinning process is a widely studied technique, there are still challenges when electrospun nanocomposites can be obtained. Direct fabrication of electrospun mats reinforced with NPs is a simple and versatile method to obtain multifunctional nanomaterials but the amount of NPs used and their possible agglomeration are still an obstacle to in depth study. Among all the inorganic elements, Mg-based NPs show a great variety of advantages for different applications with, in the last decade, MgO NPs being widely used in electrospinning researches. In particular, special attention is being paid to the catalyst ability of these NPs for industrial applications. However, no many studies are still reported based on other Mg-based NPs, such as Mg(OH)_2_ or pure Mg NPs. 

The main potential applications of woven no-woven electrospun nanocomposites reinforced with Mg-based nanoparticles have been summarized taking into account different fields of applications very present-day and important as for environmental assessments such as waste-water cleaning and air filtration, energy devices, catalysis as well as for novel technical textiles. However, the mayor application of Mg-based electrospun materials is in the biomedical field, as pointed out throughout this review, due to the osteoconductivity, osteoinductivity, and antibacterial properties of the Mg-based NP, among others. In tissue-engineering applications in particular, Mg-based electrospun nanocomposites show promising results since their mechanical properties improve with respect to the neat polymer mat. In addition, cellular growth is found to be suitable on these materials together with their antibacterial activity against Gram-positive and Gram-negative bacteria. 

The perspectives for using electrospun materials based on Mg nanoparticles are encouraging, including the use of Mg-based nanoparticles with biodegradables polymers. However, although many technical problems still need to be improved, the research works revised in the present review clarify the promising tendency in using electrospinning technique for huge development of Mg-based reinforced materials at the industrial level in the near future. 

## Figures and Tables

**Table 1:** Electrospinning parameters and their effects on the fibers formation.

**Polymer Parameters**	**Effect on the Fibers**
Molecular weight	With an increase in molecular weight, the number of drops and beads decrease.
Polymer concentration	With an increase in polymer concentration, the average diameter of fibers increases.
Viscosity	With an increase in viscosity, the number of drops and beads decrease and the average diameter of fibers increases.
**Processing Conditions**	**Effect on the Fibers**
Voltage	With an increase in voltage, the average diameter of fibers increases.
Flow rate	With an increase in flow rate, the average diameter of fibers increases.
Collector	Changing the collector type, aligned or random fibers can be obtained.

**Table 2:** Summary of electrospinning parameters, nanocomposite properties and activity against target adsorbate.

Polymer	NPs	Electrospinning Parameters	Post-Electrospinning Process	Fibers Diameter (nm)	Adsorbate	Performance *	pH	Ref.
Cellulose acetate-Poly ethylene oxide	MgO	V = 16–18 kV, D = 15 cm, Fr = 0.04–0.08 mL/h.	Direct	1350 ± 390	Methyl parathion	33%	–	[82]
Poly ethersulfone	MgO	V = 18 kV, D = 15 cm, Fr = 2 mL/h.	Surface treatment	–	Ethyl Paraoxon	92%	–	[83]
Magnesium Acetate–Magnesium Citrate	–	V = 20 kV, D = 20 cm, Fr = 0.6 mL/h.	Calcination	250–500	Fluoride Congo Red	237.49 mg/g 4802.27 mg/g	–	[84]
Poly vinyl alcohol	MgO	V = 26–28 kV, D = 10 cm, Fr = 0.5 mL/h.	Calcination	35–200	Remazol Yellow	98%	–	[85]
Poly vinylpyrrolidone	MgO	–	Calcination	180–260	Uranium (VI)	90 mg/g	6	[86]
Poly acrylonitrile	MgO	V = 12 kV, D = 20 cm, Fr = 1 mL/h.	Calcination	316.6 ± 41.72	Cadmium (II)	3 mg/g	–	[87]
Poly propylene glycol	MgO	V = 21 kV, D = 15 cm, Fr = 0.5 mL/h.	Calcination	60–76	Lead (II) Copper (II) Cadmium (II)	1900.05 mg/g 1919.28 mg/g 1922.79 mg/g	7.5 7.5 7.5	[88]
Poly vinyl alcohol	MgO	V = 15 kV, D = 20 cm, Fr = 1.0 mL/h	Calcination	1230 ± 340	Lead (II) Copper (II) Methylene Blue Fulvic acid	753.1 mg/g 481.0 mg/g 315.6 mg/g 24.0 mg/g	6–7	[89]
Poly amide 6	Mg(OH)_2_	V = 20 kV, D = 15 cm, Fr = 0.5 mL/h	Hydrothermal process	–	Chromium (VI)	296.4 mg/g	2	[91]
Poly acrylonitrile	MgO	V = 15 kV, D = 13 cm,	Direct	31.33–116.94	Dioctyl phthalate particles	99.97% for collecting the 0.3 µm particles	–	[93]

* Performance measured as mg of adsorbate per gram of material or percentage.

**Table 3:** Requirements for the design of scaffolds in bone tissue engineering [141,142,143,144,145,146,147].

**Indispensable Requirements**	**Importance of the Requirement**
Biocompatibility	Capacity to be in a host tissue without initiating an inflammatory response.
Osteoinductivity	Ability to recruit and differentiate mesenchymal cells.
Suitable chemistry	To allow protein adsorption between implanted scaffold and surrounding tissue.
Suitable surface topography	Influence cellular behavior such as adhesion, proliferation, and differentiation.
3D structure	To host the newly formed tissue.
Mechanical properties	To support the defect area. Influence cell behavior.
Porosity and pore shape	To allow tissue ingrowth, nutrient and oxygen change; neovascularization and influence cell behavior.
Wettability	A proper wettability enhances the adhesion of proteins and thus the cell attachment.
**Desired Additional Requirements for the Design of Scaffold in Bone Tissue Engineering**	**Importance of the Requirement**
Antibacterial activity	To inhibit the growth of bacteria and prevents infections.
Advanced smart properties	To respond to an external stimulus (Shape Memory Polymers, piezoelectric capacity, drug reléase, …).

**Table 4:** Characteristics of synthetic and natural polymers reprinted from ref. [158].

Characteristics	Synthetic	Natural
Polymer Types	Poly(anhydride), Poly(propylene fumarate) (PPF), Poly(caprolactone) (PCL), Poly(phosphazene), Poly(lactic acid) (PLA), Poly(ether ether ketone) (PEEK) poly(glycolic acid) (PGA) poly(lactic-co-glycolic acid) (PLGA)	agarose alginate collagen fibrin, glycosaminoglycans hyaluronic acid, chitosan silk
Advantages	inert, high reproducibility, availability on demand, reduced costs, constant quality supporting industrial scale production, possibility to design or tune, mechanical properties, composition adaptable to needs, possibility to fabricate complex shapes, controlled degradation rate, long shelf life, cell attachment improvement, potential to deliver soluble molecules	readily available, mass producible, large quantities constantly available, cost, low immunogenicity, bioactive properties, binding sites for cells and adhesion molecules
Drawbacks	immune response, lower ability to interact with cells, strong inflammasome reaction	sterilization cost, in vivo source natural variability, lot-to-lot variability, limited mechanical properties, degradation rate difficult to control, unwanted immune reactions due to impurities
Host Innate Immune response	high	low
Host Adaptive Immune response	not applicable	low

**Table 6:** Summary of electrospinning parameters used, mechanical properties of biocomposites and their main biomedical applications.

Polymer	NPs	Electrospinning Parameters	Post-Processing	Fibers Diameter (nm)	Properties	Applications	Ref.
PCL-Keratin	MgO	V = 11 kV, D = 10 cm, Fr = 1–2 mL/h.	Direct	200–2200	E: 1–10.5 MPa UTS: 0.5–3.5 MPa	Tissue engineering, drug delivery, wound healing	[183]
Alginate-PVA	MgO	V = 26–28 kV, D = 10 cm, Fr = 8–10 µl/h.	Direct	60–250	UTS: 4.5 MPa EB: 6.73%	Regeneration of tissues, extracelular matrix substitutes	[185]
PCL	MgO	V = 19 kV, Fr = 1.9 mL/h.	Direct	200–600	E: 16.8 MPa UTS: 7.3 MPa	Bone–soft tissue engineering scaffold	[186]
PCL-CS	MgO	V = 25–27 kV, D = 7 cm, Fr = 2.5 mL/h.	Direct	60–250	UTS: 2.3–2.6 MPa E: 6.8–8.6 MPa	Tissue engineering, extracelular matrix substitutes	[187]
Poly (vinyl pyrrolidone)	SiO_2_-CaO-MgO-P_2_O_5_	D = 6 cm	Calcination	156	-	Bond formation, bone substitutes	[182]
PCL	SiO_2_-Na_2_O-K_2_O-MgO-CaO-P_2_O_5_	V = 17 kV, Fr = 1 mL/h,	Deposition of polymer fibers on bioactive glass fibers.	20000 ± 2300glass 750 ± 540PCL	UTS: 60 ± 16 MPa EB: 10 ± 2%	Nerve growth, nerve guide	[184]
PLA	Bioglass with MgO	V = 16 kV, D = 20 cm, Fr = 0.15 mL/min.	Direct	3300–6900	-	Bone tissue regeneration	[188]
Poly urethane	MgO	V = 10 kV, D = 20 cm, Fr = 0.2 mL/h.	Direct	From 622 ± 174.75 to 522 ± 159.10	UTS: 8.18–10.15 MPa EB: 320–400%	Regenerative medical applications	[189]
PCL	LDHs	V = 20 kV, D = 20 cm	Direct	600 ± 50PCL-LDH 350 ± 50PCL-LDH organically modified	-	Host of active molecules, drug molecules exchange	[190]

NPs (Nanoparticles), V (Voltage), D (Distance), Fr (Flow rate), E (Young modulus), UTS (Ultimate tensile strength), EB (Elongation at break)
